# Addressing environmental harms in the health sector: environmentality as a lens to expose (neglected) sites of knowledge/power

**DOI:** 10.1057/s41599-025-05307-8

**Published:** 2025-07-01

**Authors:** Gabrielle Samuel, Stephen Roberts

**Affiliations:** 1 Department of Global Health and Social Medicine, King’s College London, London, UK; 2 Institute for Global Health, University College London, London, UK

**Keywords:** Health humanities, Environmental studies, Sociology

## Abstract

In an era of increasing calls for responsible environmental stewardship within health research and care, the concept of environmentality is a productive vehicle to theorise, analyse and critique the changing trends of environmental governance. Despite the usefulness of this approach, little to no literature has explored how this concept could apply to the health sector. In this paper, we examine three examples of emerging environmental governance in the health sector to illustrate and consider the usefulness of the environmentality lens. We show how environmentality provides a framework to interrogate different forms of governance and, in particular, how specific modes of environmental governance gain traction such that different types of knowledge/power (relations) are produced. We argue that using this analytical framework can draw attention to the regimes, techniques and technologies that are beginning to shape the forms of knowledge that are gaining power in health sector environmental management and can contribute to a better understanding of fields of environmental knowledge/power (in)visibility.

## Introduction

The health sector contributes to 4–5% of global greenhouse gas emissions, and to a range of environmental harms, including the production of both toxic and non-toxic waste, and the use of water and other resources (Hodges, 2017, Pichler et al. 2019, Lenzen et al. 2020). To address these harms, 143 countries have endorsed a declaration to promote ‘steps to curb emissions and reduce waste in the health sector, such as by assessing the greenhouse gas emissions of health systems, and developing action plans, nationally determined decarbonisation targets, and procurement standards for national health systems, including supply chains’ (COP28UAE, 2023). The Alliance for Transformative Action on Climate and Health (nd), a group of 80 countries, has pledged to reduce the greenhouse gas emissions of their health systems, and similar pledges have been made in the health research sector (Weber et al. 2024). The World Health Organisation’s ‘Operational framework for building climate resilient and low-carbon health systems’, published in November 2023, provides guidance to achieve these emissions reductions, and funding bodies are increasingly funding health research into environmental sustainability (World Health Organisation, 2023). In the United Kingdom (UK), where the authors reside, the National Health Service (NHS) of each devolved nation aims to advance Net Zero policies by between 2030 and 2050 (depending on the devolved nation) (NHS, 2020), and these commitments are backed by two pieces of legislation: the UK’s Climate Act 2008^1^ and the Health and Care Act 2022.^2^ In a similar vein, the UK’s research and innovation sector, which includes health research, has co-developed a voluntary environmental sustainability Concordat (2024) to represent a ‘shared ambition for the UK to continue delivering cutting-edge research, but in a more environmentally responsible and sustainable way’. The Concordat is housed on the website of the Wellcome Trust, a leading national and international health research funder. Alongside such initiatives, advocacy work and the development of best practice guidance is being driven at all levels (international, national, regional, local) of health research and care by health care providers, researchers, not-profit governmental organisations (NGOs), professional medical associations, and individuals (Rae et al. 2022, Roche, 2022, The Academy of Medical Sciences, 2023, Royal College of Physicians, 2024, Richie and Samuel, 2025).

As transitions towards a low-carbon, environmentally aware health sector continue, it is vital to remain cognisant that knowledge about greenhouse gas emissions (herein: emissions, meaning carbon dioxide equivalent emissions) and/or other environmental harms do not represent a fixed biophysical reality of carbon dioxide, greenhouse gas emissions, or other pollutants ‘out there’. Rather, as we demonstrate, and as an extensive and wide-ranging literature describes, such knowledge is the product of distinct socio-political processes that inform the collection of certain data types (and not others), which are then categorised in specific ways (rather than other ways), to produce a particular form of knowledge (for example, see Luke, 1995, Goldman, 2001, Agrawal, 2005, Oels, 2005, Hart, 2011, Jepson et al. 2012, Lippert, 2015, Wang, 2015, Peck, 2016, Whitington, 2016). As Luke (1995) maintains, the environment must therefore be understood not as the naturally given sphere of ecological processes, but as a historical artefact that is openly constructed through the generation of knowledge. Without this understanding, meanings of the ‘environment’ as used in health sector environmental governance may potentially be conflated with the biophysical environment, such that the limitations of such meanings are not interrogated in practice.

Little to no attention has focused on this socio-political construction of *environmental* knowledge in the health sector despite ideas and understandings associated with social constructions of knowledge being well established in health research and care more broadly, with scholars long having extensively explored how socio-political factors shape the production of health related knowledge (for example, see Cetina, 1999, Brown, 2003, Borup et al. 2006, Jasanoff and Kim, 2009, Jasanoff, 2015). This lack of attention is a disservice: as with any form of socio-politically constructed knowledge, constructed environmental knowledge will inform certain governance modes, such that the knowledge produced will render the environment governable in a particular way, and through this knowledge, legitimise and justify management through specific strategies of power (Luke, 1995, Goldman, 2001, Agrawal, 2005, Oels, 2005, Foucault, 2007, Hart, 2011, Jepson et al. 2012, Wang, 2015). Knowledge and governance are therefore intricately tied; certain types of knowledge will inform governance decision-making processes that will have certain types of implications. In other words, understanding how the environment becomes knowable allows us to critique and interrogate the specific knowledge/power configurations associated with environmental governance, and, within these, the knowledge/power dynamics associated with which types of knowledge come to be prioritised (or made ‘visible’) over others, and how this knowledge is leveraged, reproduced and enacted through specific modes of governance. As Hart (2011: 31) writes, it allows us to explore ‘how certain aspects of it are constructed as “truth”, how technologies and experts are constructed around it, and how subjects are formed based on these analytics’. This is important because specific knowledge will lead to governance models that will generate particular and impactful effects, including both intended and unintended consequences—what Tenner (1997) elsewhere has called ‘revenge effects’.

Together, the above underscores the need to cultivate a broader and more nuanced understanding of how the environment comes to be understood in the health sector as an object and target of governance via distinct socio-political processes and knowledge/power dynamics. Undertaking such an analysis can expose the limitations of specific modes of environmental governing to help foster a more appropriate governance system for sustainable and equitable environmental governance practices in the health sector.

In this paper, we analyse the usefulness of an *environmentality* lens as a vehicle to understand and assess how specific modes of knowledge production gain power through informing environmental governance responses in the health sector. The foundational precepts of environmentality are drawn from Foucault’s well-known theories of governmentality and bio-power (Foucault, 2007); in fact, the *environmentalit*y lens has also been termed ‘green governmentality’ and ‘eco-governmentality’ (Malette, 2009, Valdivia, 2015, Rutherford, 2017). Each of these terms presume the socio-political construction of the concept of the environment and then take this perspective to explore the knowledge/power dynamics associated with environmental governance.

We specifically use *environmentality* rather than *governmentality*, even though the latter is extensively used in health literature, because while governmentality can serve as an analytical lens on the environment, it mainly focuses on the knowledge/power associated with the governance and health of populations (Foucault, 2007). Environmentality brings the governmentality approach to study the non-human environment: it sees the object of knowledge to be governed as that which is related to environmental knowledge construction rather than the construction of knowledge about specific individuals, groups or populations. Taking this approach therefore allows us to shift away from exploring the governance of populations (bio-power), to theorise, analyse and critique the governance of environmental harms through a knowledge/power perspective, or what Luke (1995) calls ‘geo-power’. In essence, the concept of environmentality allows us to approach environmental governance as ‘a site of power, where truths are made, circulated, and remade’ (Rutherford, 2017: 1). Working with this lens, we can expose how power/knowledge about environmental problems circulates, which governance configurations are produced in response, and what consequences emerge because of these configurations. These ideas allow us to challenge commonly articulated views that see environmental harms as technical problems that have neutral solutions, and to instead show how conceptions of the environment are political in nature. To our knowledge, such a perspective has not yet been applied in this field.

To achieve our aims, this paper is structured into three sections. First, we elaborate the concept of environmentality, and how modes of knowledge/power are enacted and presented through various techniques, institutions and discourses to shape and regulate the conduct of individuals and populations through environmental governance. Second, we draw on three examples of environmental governing within the health sector to illustrate the benefits of using this theoretical framework to better understand how knowledge/power is operating and the implications that arise from this. Third, we reflect on these examples and the usefulness of applying an environmentality approach in the health sector.

## Environmentality

Environmentality is a theoretical framework for analysing the increasingly complex global landscape of environmental imaginaries, governance and regulation. It is informed by Foucault’s concept of governmentality, which has been extended by many in the years since his death in 1984. As presented by Foucault, governmentality is a theoretical framework to understand the function and influence of governance and power, and how power operates and functions through specific channels within the modern state. A central premise is that power is not necessarily negative or oppressive; instead, it is concerned with the regulation and governance of populations to ensure their compliance, productivity and health (1991). This is accomplished, according to Foucault, through the prescription of specific policies and practices, shaped by knowledge and enacted through systems of power. Governmentality is aided through an ‘ensemble of institutions, procedures, analyses and reflections, calculations and tactics which has as its target: population, as its principal form of knowledge: political economy, and as its essential technical means: apparatuses of security’ (Foucault, 2007: 108). The focus on the population as a ‘global mass’, the technique of managing populations, and the fostering of life for the productivity of the state has become known as bio-power (Foucault, 2007: 377).

To explore knowledge/power through the Foucauldian lens of governmentality, analysis must start from an understanding of the practices of governing. Governance does not only operate in relation to spaces defined by those who do the governing but also includes the practices, techniques, calculations, methods and instruments (known as the tools of governance) that render various aspects of reality amendable to governmental intervention. Lövbrand and Stripple (2011, 190) define governance as the:

heterogeneous assemblage of mechanisms, techniques and knowledges by which the natural and social world is represented, categorised and ordered. Although these knowledge practices may seem disparate and weakly linked, they establish shared vocabularies, theories and explanations among agents across time and space. When doing so these technologies of government construct fields of visibility that shape and normalise the thought, aspirations and conduct of others.

Within governmental frameworks, knowledge is shaped and assembled by specific techniques, calculations and methods. Knowledge is not natural but rather curated and enacted to inform the conduct and self-regulation of populations, which governmentality has as its focal site of intervention and management.

Governmentality also accounts for self-governance through the ‘conduct of conduct’. Foucault explains that self-governance sits alongside state power: in the latter, power operates through direct coercion (laws, punishments); in the former, power operates through shaping how individuals and communities come to understand themselves in society (by guiding and influencing behaviour). In self-governance, individuals and/or groups regulate their own behaviour according to norms, values, discourses and systems of control that have been internalised from the state; people discipline themselves to align with societal expectations. To understand governance and power, then, we (also) need to analyse how individuals are encouraged or obliged to exercise self-government and how certain practices come to be undertaken as an aspect of the self (forms of subjectification). This can allow us to understand which aspects of being and conduct are prioritised, which aspects of individuals are rendered problematic, and what forms of knowledge are linked to these problematisations (Dilley, 2012). Ultimately, to understand how power emerges we need to interrogate governance at the level of both state and self.

Environmentality—as an extension of governmentality—‘aims to understand the ways in which governmentality can be applied to analyses of the environment and environmental governance’ (Wieckardt et al. 2022: 2616). As a theoretical framework, environmentality emerged in the early 2000s to explore how environments come to be managed in society, and to understand power in the regulation of the environment by using a Foucauldian analysis of governmentality (Malette, 2009, Valdivia, 2015). Scholars recognised that the ways the environment was being understood in global environmental politics were varied and conflicting. Some actors viewed the environment as something that required protecting, as a value in and of itself (ecocentric). In the most extreme of these approaches, deep ecologists aligned with traditional ecological knowledge and indigenous understandings of the environment, rejecting any separation between the environment and human communities (for examples, see Inglis, 1993, de La Bellacasa, 2017, Terblanché-Greeff, 2019, Jones et al. 2022). Other actors viewed the environment as a form of natural capital to be managed. Still others positioned themselves as somewhere in between these two extremes (for discussions of this middling and other positions, see, for example, Dobson, 1998, Vucetich and Nelson, 2010, Nelson and Vucetich, 2012). With such diverse understandings of the environment abounding—sometimes competing and other times overlapping—it was recognised that a conceptual approach was needed to better grapple with this complexity, the assumptions of different policy choices, and how these shaped the intended and unintended consequences of policy decisions (Fletcher, 2017). Undertaking an environmentality analysis allowed for viewing the environment as ‘an object of knowledge, about which certain “truths” can be constructed. These “truths” necessitate the regulation, management and governance of the environment’ (Wieckardt et al. 2022: 2616).

In a diverse and extensive body of scholarship, the concept of environmentality (or green/eco- governmentality) has been applied in various ways. For instance, it has been used to explore the governance of the environment to show how the environment is subsumed under the broader governance aim of protecting the health of the population through governmentality. In this view, the environment needs to be controlled as much as possible to protect humans. In other instances, scholars have shown how the environment is viewed as the entity that governs, for example, in Indigenous and relational perspectives which might view water, plants and so forth as governing the individuals and populations that live on the planet (Hart, 2011, Escobar, 2019). We focus here on probably the most used application of environmentality, that is, in analysing governance structures aimed at mitigating environmental harms and, by extension, the regulation and anticipation of future environmental risks and uncertainties. This literature is extensive, with scholars engaging with various dimensions of the governmentality literature, advancing distinct conceptual ideas and focusing on different governing rationalities.

Historically, political ecologists focused on how the concept could help explain the exertion of power through top–down approaches. Arguments centred around environmental claims-making as a means to construct notions of environmental risk that required state intervention. For example, Luke’s (1995, 1999) work, for example, places environmental movements associated with biodiversity, conservation and climate change at the forefront of generating the ‘eco-knowledge of modern governmentality’ (1995: 70). Luke views such movements as places where discourses about the environment are produced and made visible, such that this knowledge is used to govern through a form of bio- or geo-power which extended across the world (Luke, 1995, 1999; also see Wang, 2015).

As the study of environmentality expanded, scholars began to highlight other forms of top-down environmental governance. For example, drawing on Foucault’s typology of governmentalities, Fletcher (2017) proposes that environmentalities operate through four main forms: ‘disciplinary forms’ such as education, which enjoin subjects to internalise particular norms and values through which they self-regulate; ‘sovereign forms’, which use compliance via top-down injunctions backed by threats of punishment; ‘neoliberal forms’ that aim to prevent environmental degradation through the creation of market incentive structures; and ‘according to truth’, that is, truths that relate to, for instance, religious texts, of revelation, and of world order.^3^

Rutherford (2017) identifies commonalities across various approaches to environmentality, noting three interrelated areas. First is ‘the production of rationalities’, defined as the ‘forms of knowledge/power that open up particular grids of intelligibility while foreclosing others, such as statistical measurement, graphic representation, modelling, and forecasting, which all work to generate a broad picture of nature to be managed’ and emerge as indicators of risk/regulation in the modern state of environmental governance (Rutherford, 2017, 2). An example of this is Darier’s (1999) edited volume *Discourses of the Environment*, which focuses on how the mapping and measuring of the environment during management initiatives render nature into manageable categories/populations. Similarly, Agrawal’s (2005) book *Environmentality* shows how forests in India came to be mapped and measured by colonial rule to render them governable. Cullen’s (2020: 426) work would also fit here, since it argues that ‘environmental impact assessments and other environment management policies totalise state control as the singularly dominant source of authority over the environmental arena and subjects within’. The second commonality identified by Rutherford is ‘strategies of intervention’, or how various authorities—governments, NGOs, scientists, corporations and institutions—take the calculations of the first strategy, which produce knowledge that frames meanings of ‘the environment’ in a specific way, to propose solutions to environmental problems. In other words, solutions necessarily follow the knowledge provided to frame the problem. Third is ‘the generation of self-governing subjects’, such as ‘green citizens’, who are not governed *directly* through top-down systems of discipline, but rather are understood to operate freely within *prescribed* social contexts and are self-regulating and guided by power structures that encourage the internalisation of governance/power through an individualisation of responsibility (Butler, 2010, Paterson and Stripple, 2010, Rice, 2010). Soneryd and Uggla (2015) provide an example of this third commonality in their exploration of ‘the responsible consumer’, in which people are encouraged to choose ‘green’ approaches.

Beyond this useful categorisation, scholars have slowly shifted from focusing on top-down exertions of power to broader articulations of environmentality that follow how power emerges through subjectivities, which are normed and disciplined, and how subjectivities are negotiated and change through governance (Valdivia, 2015). In line with the governmentality literature, environmental governance is understood not just as happening through ‘a set of institutions’ but as ‘a practical activity that can be studied….at the level of the rationalities, programmes, techniques and subjectivities’ (Walters, 2012: 2). Scholars argue that, to explore such subjectivities and activities, we need to pay attention to people’s responses to particular forms of governance. As Valdivia (2015: 470) states, ‘governing is not force; it is an “equilibrium” between coercion and self-constitution that aims to optimise the life of populations’.

Agrawal’s (2005) work on forest conservation was seminal in this respect, showing how, when certain ideas and ideologies of conservation were brought to a local population with different views, it did not lead to the shaping of individuals’ conduct to certain ends but allowed them to transform themselves and others—their own bodies and souls, thoughts, conduct, and way of being—through active engagement. As Valdivia (2015: 474) asserts, ‘individuals have “docile” capacities that make them available to government, their bodies can be put into practice, routinised and disciplined to shape subjectivities, but they have the potential to act in other ways, too. … Power travels in multiple ways through various institutions and differently racialised, classed, and geopolitical natures, enabling multiple forms of subjectivisation’. To understand these multiple forms of subjectivisation and environmental subject-making, according to Agrawal and a multitude of other scholars (for example, see Jepson et al. 2012, Leffers and Ballamingie, 2013, Ward, 2013), attention must be paid to the entangled material geographies of belonging, exclusion, and subjugation. Such work complements the analyses of more top-down approaches to governance within environmental management.

## Environmentality in the health sector

Surprisingly, to date, very little scholarship has explored the topic of environmentality in the health sector. This is a critical oversight as an environmentality lens can shed light on the forms of knowledge/power that are shaping environmental governance, helping us better understand how specific governing modes gain traction, and in doing so, expose those knowledges that would otherwise remain invisible (Death, 2013). Exploring environmentality in the health sector is no small task. The health sector is a complex, vast conglomerate operating through different infrastructures, institutions, systems and processes, and different instances and types of environmentality are likely to operate through distinct contexts, places and sites of practice. A comprehensive account of the various forms of environmentality circulating in this arena is thus beyond the parameters of this paper. Rather, our aim is more modest: to apply a lens of environmentality to three instances of environmental governance in the UK health sector to illustrate and consider what such a lens can offer. The instances include (a) carbon accounting for health practices and processes, (b) promoting the use of the ‘green inhaler’ and (c) developing regulations to govern environmentally sustainable health research. Each of these represents one of Rutherford’s (2017) three interrelated areas of environmentality scholarship. In describing these instances through an environmentality lens and by focusing on issues of knowledge production and power, we show how power configurations enable the environment to be rendered thinkable and governable. In doing so, this allows us to offer a critique of the seemingly self-evident (Lövbrand and Stripple, 2011).

First, we describe carbon accounting as an example of Rutherford’s (2017) ‘production of rationalities’, or the first stage of governing that opens up particular ways of knowing the world (while foreclosing others) by creating regulation indicators to be used in governing. In the case of carbon accounting, a specific rationality is produced by quantifying biophysical greenhouse gas emissions and entering them on a spreadsheet for quantitative data-intensive analysis of carbon-emission equivalents, and in doing so, producing new specific forms of knowledge (Lippert, 2015, 2018, Peck, 2016, Whitington, 2016).

Once carbon accounting has opened up this particular way of knowing, it produces specific rationalities of governance, to which our second instance of ‘green’ inhalers relates. This instance temporally follows the production of this new accounting knowledge—and the meanings of the environment associated with it—and how it brings power to certain governance modes. In this example, we see that carbon accounting has led to the implementation of governance approaches that promote the use of low-emission green inhalers, while paying less attention to other inhaler-associated environmental harms and impacts. Furthermore, we show how the framing of knowledge about inhaler-associated emissions has shaped who is deemed responsible for reducing such emissions, with responsibilities being attributed, through self-governance, to the patient.

The third instance examines the field of UK health research to illustrate how knowledge about environmental harms associated with specific practices does not simply lead to governing power through the implementation of top-down governance, but rather these interventions are a site of negotiation for multiple subjectivities, with meanings of environment being shaped by various interests (Valdivia, 2015). As these new regulations gain power, new knowledge is produced, and this power/knowledge spreads across the sector through new forms, tools and modes of governance, expanding by means of self-governance rationalities and practices. Tools designed to survey awareness in the field unwittingly further extend and contribute to the generation of this new knowledge.

### Carbon accounting

To govern the environment, we must first render it as something that is problematic and in need of governance. In recent years, due to the urgency of climate change, the governance of greenhouse gas emissions has become a prominent example of environmental governance. The goal of this governance is to measure carbon-emission equivalents with the aim of then reducing them to a number. In fact, over the last decade or so, there has been a massive proliferation of carbon-measurement devices and carbon-auditing methods to assist individuals, organisations and institutions with such assessments.^4^ These approaches have been extensively researched by social scientists, who argue that such processes reduce greenhouse gases to a set of categories and numbers, based on subjective assumptions and value judgments (Paterson and Stripple, 2010, Lippert, 2015, Peck, 2016, Whitington, 2016, Lippert, 2018). Walenta (2021: 533) explains, the ‘carbon footprint serves as an impact measurement device that translates the complexity of carbon molecules’ behaviour in the atmosphere into quantifiable discrete units in a spreadsheet’. The use of categories and numerical management enabled by carbon accounting echoes the earlier work of Hacking (1982), who described how the ‘avalanche of printed numbers’ in the modern era gave rise to systems of statistics and quantification that allowed the world to be categorised, represented, understood and managed. Hacking’s analysis builds upon earlier discussion of the emergence of statistics in the modern era and how it constituted new forms of knowledge that informed the exercise of power and the identification of risk. Below, we explore how carbon accounting constructs this type of knowledge.

#### Measuring carbon, carbon quantification and carbon calculators

As measuring carbon emissions relies on the collection of data, a fundamental problem in quantifying carbon emissions is the availability of data upon which to base calculations. Some data is available online. For example, respected organisations, such as the Intergovernmental Panel on Climate Change,^5^ publish tables to provide conversions to carbon-emission knowledge for select products and/or processes. Often, however, these conversions do not include the necessary information or the amount of detail required for analysis. In addition, all approaches to carbon accounting require accessing data from other individuals, organisations and/or institutions, some of which is easier to access than others (for example, private sector companies may choose not to disclose such information).

A range of methodologies has been developed to assist with calculations, with some more detailed than others. Whatever approach is chosen, however, considerable time is required to develop the calculations, including deciding what to count and how, and which parameters to use. Parameters are often related to what data are accessible, as well as what is feasible to measure (Nowotny et al. 2001, Freidberg, 2018). Once data has been collected, it is inputted into spreadsheets, which allow the data to be processed, categorised and modelled in various ways. This involves ‘cleaning the data’—fixing or addressing incomplete, incorrect, duplicate or incorrectly formatted data—and reformulating for analysis, the outcome of which shapes the type of knowledge produced, disseminated and available for governing regimes.^6^

Carbon quantification, then, is not a fact-finding mission to obtain some ultimate objective truth, nor is there a ‘correct’ scientific way to quantify and calculate carbon emissions. Rather, it is an iterative sociological representation of a phenomenon, driven by data collection and quantification, and enacted via processing, spreadsheets, data cleaning and software. Accounting is always approximate, using a modelling process and varying degrees of precision, depending on how the assessment is framed and conducted. The scope of calculations will shift depending on the methodology, with different approaches relying on different assumptions based on different bodies of knowledge. The accessibility of data also plays a role in choosing methods, which data to include (or not) in the calculation, and how to do so. For instance, when calculating carbon emissions associated with the manufacture of a particular device, the further upstream a researcher travels to assess these emissions, the harder it becomes to decipher how many of the upstream emissions are associated with the downstream device. This is because each device component is one small percentage of an upstream process that has manufactured components for multiple devices. Researchers must resort to a certain amount of estimation in their analysis (for a discussion of this in the digital sector, see Samuel et al. 2024). Furthermore, the wider the research question, the more indeterminate and uncertain the figures; the narrower the analysis is, the less chance of capturing all of the emissions in the calculations (Samuel et al. 2024). In an example from the digital sector, some researchers include the use of televisions as digital devices, while others do not - see, for example, Malmodin et al. (2024) for an instance in which televisions and other media are excluded. Decisions will sometimes be driven by practicality and/or feasibility, while at other times they may be more related to political interests (a fact that underlines the importance of always noting who or what institution is conducting the study).

Finally, because the data used during carbon calculations are sourced from other individuals, organisations and/or institutions, each drawing on their own metrics and methodologies, certain assumptions will underpin the data. These might include, for example, parameters used to develop data models at particular time-points that are likely to change over time, such as the energy mix of specific electricity grids in particular countries; the country in which products are produced and/or processes occur, and how this affects their associated carbon emissions; and the changing efficiency of certain processes over time. Such assumptions are often not recognised or understood by those using the data for their own analysis, who are not experts in the field from which the data is obtained (Collins 1981, 1999). As Collins and others have explained, scientists tend to ‘black box’ areas of controversy and uncertainty, so those close to a particular field of science are often aware of the uncertainties of the methods, but ‘those distant from the research front, and thus not exposed to the art and craft of scientific practice, get a view of science relatively free of doubts and uncertainties’ (Collins, 1997: 165).

#### Carbon accounting in the health sector

In the health sector, carbon-auditing processes and calculators are increasingly being used to quantify greenhouse gas emissions (as carbon-emission equivalents). Some carbon calculators are relatively easy to use, for example, based on software developed to input numbers. Greenalgorithms—a calculator designed to assess carbon emissions via machine-learning algorithms—is an example of this.^7^ It allows a researcher to input the location of analysis and the model of the programme being used, and offers a variety of visualisation tools to understand the findings. In other instances, carbon auditing requires specific statistical expertise, leading to the creation of new fields as actors enter these spaces to develop new methods and implement standard methodologies to support the expansion of carbon auditing to more practices and processes. The greener clinical trials methodology is an example of this. Given the complexity of processes involved in conducting clinical trials, the methods designed to assess their carbon emissions require a certain amount of training, and networks have been established to support such training through drop-in sessions, documents, webinars and conference presentations, for example, the UK MRC-NIHR TMRP Greener Trials Working Group Remit, which is part of the Medical Research Council Hub for Trials Methodology Research.^8^ Other examples include the Health Care Emissions Calculator,^9^ the Care Pathway Carbon Calculator,^10^ the Climate Impact Checkup^11^ and Practice Green Health’s carbon calculator.^12^ Further still, open-access databases containing environmental-impact assessments of various health-related practices and processes have been developed to help researchers in their calculations (Drew et al. 2022).

The number of training schemes, guidelines, conferences and seminars to support interest holders in carbon accounting skills has grown dramatically (for example, see the UK’s Centre for Sustainable Healthcare).^13^ Research funding calls are being increasingly announced to drive research into approaches to decarbonising the health sector (NIHR, 2014). These developments generate more statistical knowledge about carbon accounting and more trained individuals, leading to an expanding network of skilled actors who reproduce methodological approaches and training in these spaces. In other words, this rise of activities, events, training and information sources around carbon-calculation leads to an expansion of new networks of knowledge-making associated with carbon accounting, forming ever larger environmentality frameworks around this form and type of knowledge.

Lövbrand and Stripple (2011) maintain that enacting a rationality of governance through carbon accounting gives way to new domains of political calculation, such that measuring carbon emissions is synonymous with power: if we know, we can act. We see this in the health sector as advocates call for changes to practices within specific modes of environmental governance based on the accumulation of carbon-accounting knowledge. Carbon emissions associated with various health-related processes have been assessed to inform decisions about the best course of action, both in healthcare operations, such as laundry (John et al. 2024), as well as in care pathways, such as surgery (Thiel et al. 2018). Bhopal and Norheim (2021), who argue that policymakers must include carbon emissions when evaluating healthcare interventions, provide examples of the estimated carbon costs and health benefits for four interventions (emergency caesarean section, laparoscopic hysterectomy, cataract surgery and robot-assisted prostatectomy) in high-income countries to illustrate how much health a tonne of carbon can buy (Bhopal and Norheim, 2021). Political change is happening within the UK health sector. For example, the NHS sustainable procurement plan requires that by 2027, ‘all suppliers will be required to publicly report targets, emissions, and publish a carbon reduction plan for global emissions’.^14^

From this analysis, we can see how, through various calculative practices, the social construction of carbon is hidden in the health sector, replaced by views that constitute carbon as a stable object that can be collected, measured and acted upon. In essence, the types of knowledge produced via carbon accounting, which are neither neutral nor ephemeral, are translated into and inform a range of political actions around the governance of the environment and governance techniques. In this way, carbon accounting acts as a site of knowledge/power nexus.

While the rise of carbon accounting in the health sector has enabled a certain static understanding of carbon and its measurement to take hold within environmental governance spaces, there has been limited critical discussion of how these knowledge/power practices simultaneously obfuscate alternative methodologies, narratives and imaginaries of environmental knowledge and practices. This has the effect of narrowing not only environmental understandings but also avenues for potential diverse participation and knowledge transfer in collective climate action. It is here to which we now turn.

### Green inhalers, strategies of intervention and the generation of self-governing subjects

As discussed in the introduction, globally, healthcare has been estimated to account for 4–5% of emissions; high-income settings such as the UK typically report higher levels (Karliner et al. 2020). Governance initiatives to drive reductions in the UK health sector include state legislation (for example, the UK’s Climate Act 2008 and the Health and Care Act 2022);^15^ sector approaches (see, for example, the establishment of the UK’s Greener NHS); regional initiatives, such as local hospitals instigating low-emission transport-to-work schemes (NHS England, 2023); and switching to care pathways that produce fewer emissions. In terms of the latter, the NHS has flagged the use of inhalers in respiratory care as important because these devices account for ~3% of the NHS total emissions (NHS, 2020). Most patients use metered-dose inhalers that contain hydrofluoroalkane gases, which have a Global Warming Potential thousands of times greater than carbon dioxide. Low-emission dry-powder inhalers (‘green inhalers’) are available and do not contain these gases. For many patients, dry-powder inhalers can offer clinically equivalent treatment without the high emissions associated with metered-dose inhalers, though they are not appropriate for all patients (for example, the young, the elderly, those who struggle with breathing) because of the skill required for use. The NHS is working to reduce inhaler-related emissions by encouraging physicians to have conversations with their patients about switching inhalers so that patients can then make the most appropriate decision for their care. In the below we show, first, how this approach has shaped a particular mode of governance that emphasises patients as self-governing, and second, how this approach has led to particular meanings of the environment being governed, prioritising certain forms of knowledge (carbon-emissions equivalents), and hiding other forms of knowledge about environmental harms. We take each in turn.

First, the NHS’s approach places patient choice at the centre, framing physicians as key providers of knowledge/power for reducing inhaler-related emissions, and patients as the site of governance to manage risk. As Parker and colleagues (forthcoming) show, knowledge/power is provided to patients, driven and encouraged by discourses communicated from their doctors, so that as self-governing individuals, they can make their own choices about inhaler-related risks and harms. A vast array of patient-related information and literature has proliferated in the form of pamphlets and leaflets to assist patients with making such choices, including informing them about the benefits and risks, and opportunities and limitations associated with using/switching inhalers, as well as descriptions of those patients best suited to using low-emission inhalers, and those who should avoid them. Information/knowledge also directs patients on how best to use low-emission inhalers, which require specific respiratory skills, to ensure their clinical effectiveness (NICE, 2022, Greener Practice n/d). An example of such information is found on the Greener Practice website, which states that:

Some inhalers have a much higher carbon footprint than others so you may want to review your inhalers. … The right inhaler for you is the one you will use correctly. … If you are concerned about the possible environmental impact of your inhalers it is very important that you discuss this with your doctor or nurse, rather than just stopping your inhalers.^16^

In this extract, environmentality operates at the level of the patient; describing patients who ‘want to review their inhaler choice’, the text evokes such patients as ‘good citizens’. This specific patient-focused, risk-based knowledge/power is complemented by knowledge/power from ethics scholars who are beginning to focus on the ethical considerations associated with the switch to green inhalers, thereby further making inhaler switching visible as a mode of governance (Health Systems Global, 2024). Seen through an environmentality lens, the communication and positioning of knowledge occurring at the interface of doctors and their patients, focused on the promotion of uptake of green inhalers, further gives way to and enables a form of environmental management that not only is predicated on exclusive forms of knowledge but also elevates the role of individual agency and responsibility in environmental management initiatives across healthcare sites including within clinics and consultation rooms. Here, patients, provided with counsel and knowledge from medical authorities, are therefore enabled to consider and select their healthcare choices in alignment with broader concerns for the environmental impact of these interventions, producing new self-governing subjects within environmentalities of the healthcare sector.

Second, this framing brings carbon-centric knowledge/power to environmental management, with other knowledge/power aspects being ignored or underexamined. As Parker (forthcoming) argues, although dry-powder inhalers have a significantly smaller carbon footprint than metered-dose inhalers, other aspects of their production and disposal are damaging to the environment. Jeswani and Azapagic’s (2019) life-cycle assessment compares the environmental impacts of dry-powder inhalers with metered-dose inhalers to show that although dry-powder inhalers have lower associated emissions and effects on ozone depletion, they have a worse impact on other ecological concerns, such as marine eutrophication, fossil depletion and photochemical oxidant formation. Furthermore, while emphasising the importance of using dry-powder inhalers, Murphy and colleagues (2023) write that dry-powder inhalers are more difficult to recycle than metered-dose inhalers, as these inhalers are classified as ‘non-recyclable’ in the Chiesi inhaler recycle scheme.

This example of green inhalers demonstrates how narrow conceptions and framing of both the environment and approaches to environmental management can lead to further environmental degradation. While carbon-centric framings have presented dry-powder inhalers as greener health interventions in the management and regulation of the environment, further research has, by contrast, highlighted the negative environmental impact of dry-powder inhalers associated with their production, life cycle and disposal. This again indicates the need for further continued critique and assessments of which channels of information and knowledge are brought to the surface within environmental frameworks, and of how unchallenged implementation of such knowledge might further impact environmental sustainability.

### Environmentally sustainable health research and strategies of intervention

In the UK and more widely, and largely driven by a bottom-up advocacy push, there have been shifts to make health research more environmentally sustainable, including recommendations for researchers and research managers to reduce emissions associated with their practices (Lannelongue et al. 2021, Souter et al. 2023),^17^ as well as accreditation systems to guide researchers through these emission-mitigating practices (Sustainability Exchange n/d). These shifts are part of a broader effort to bring environmental governance into research institutions (Gormally et al. 2019). Most recently, as described in the introduction, a UK Concordat for Environmentally Sustainable Research has been developed and published, and, in the health sector, UK health research funding bodies are piloting assessment criteria, including carbon calculators and narrative assessments, as a way to environmentally govern research through top-down approaches (for example, see MacFarlane and Samuel, 2022).

Top-down governance productively disperses power because it creates an apparatus for power/knowledge production through metrics and/or evaluation requirements, which then go on to shape practices and further knowledge production. In fact, our own studies in the field of data-intensive/digital health research show that researchers felt unsure about how to self-govern in terms of addressing the environmental harms associated with their research, and sometimes struggled to reconcile how to be responsible in practice when managing other priorities (Samuel, 2023; also see Gormally et al. 2019); they felt that the imposition of top-down regulation would be the most productive way to guide their practices. In essence, they wanted to *defer* some of their responsibility to funding bodies, take on the role of *obedient subjects*, and ask funding bodies to leverage their power to require them to enact change. They were happy to enact this change because they felt a sense of responsibility and obligation to consider the environmental harms associated with their research, in line with broader societal norms that have constructed environmental issues through the lens of individual responsibility, what Rose (1999) has called the ‘responsibilisation of the individual’ (also see Soneryd and Uggla, 2015).

However, researchers were only amenable to top-down governance if it did not dramatically affect their practices and research agenda (Samuel, 2023). Funding bodies, which have been key drivers of such top-down regulation, have accommodated this preference through a range of consultation practices and pilot schemes to help develop new modes of governance. Potential governance schemes have thus become a site of negotiation regarding the meaning of the environment in the research endeavour. As such, while the researchers described their reflections on being governed in a way that can be interpreted as ‘docile’ capacities that make them available to government (Valdivia, 2015), they displayed active modes of subjectivisation, pushing back against certain meanings of the environment to create environmental governance that was in line with their own preferences. For example, some insisted that funding bodies should engage with health researchers before disseminating any governance tools into practice. Consultations, iterative refining of tools based on researcher feedback, and pilot schemes are all key processes being implemented to ensure that funding body regulations and requirements construct a governing space within which researchers have helped construct meanings and practices. What becomes acceptable, and therefore legitimised, will be an outcome of this process, and inevitably then a further important site of knowledge production and power, as new knowledge related to these requirements is produced, circulated and enacted. Here we emphasise that this interplay is not necessarily negative. As discussed earlier, Foucault emphasised how power is not to be understood as repressive but can also operate constructively and can inform understandings and organise and guide action. Analysing these processes of knowledge co-production within health research through the lens of environmentality is therefore not to criticise oppression but rather enables an understanding and profile of key agents of knowledge/power, modes of subjectivisation and resistance, and the forms of knowledge which enable governance generated by these dynamics.

Finally, environmentality can productively expose how researchers themselves act as sites of knowledge making. For example, as the pace for considering environmentally sustainable practices in health research gains power, a range of surveys have aimed to assess researchers’ awareness of the environmental impacts of their health research (Samuel, 2023).^18^ Other surveys have been designed to assess environmental behaviour and the barriers to implementing environmentally sustainable practices,^19^ and webinars and training opportunities are increasingly offered to build awareness about the environmental harms associated with health research. Through an environmentality lens, we can see how, together, these tools act as a site of knowledge/power by having a responsibilising effect that drives individuals towards self-governance through environmental socialisation (Hargreaves, 2008)., i.e., surveys which interrogate awareness associated with environmental harms of health research also have the dual effect of providing awareness, which in turn, suggests a need to act (moralising, responsibilising, socialising effect). In a similar way, it has become increasingly acceptable for researchers to use carbon calculators to monitor the carbon emissions associated with their own research activities. In doing so, researchers are unwittingly converted into both the gatherers and providers of data, which can then be used to support and shape a particular form of environmental knowledge/power which further shape and inform political action which discursively frame and govern these health researchers and the environmental risks of their practices (Gabrys, 2014). As such, knowledge/power does not just lead to the production of tools to be used in top-down governance approaches for health research’s environmental governance, but governmentalities emerge from the practices of researchers who provide data through these new carbon-calculation technologies (Gabrys, 2014).

## Environmentality as a useful concept

Returning to the work of Foucault, and as the case studies within this paper have illustrated, different types of environmentalities usher forward different modes of governing populations within environmental spaces, extending governmentality into new zones of government and intervention. The case studies are also useful in charting the expansion of new environmental governance practices that operate through modes of self-governance and technologies of the self, as Foucault (1998) described them, whereby individuals self-regulate and enact choices and practices via internalised understandings about how they should behave. In the case of environmentality, this self-regulation happens around the provision of health services and within green initiatives within the healthcare sector via internalised understandings of the environment brought into existence by specific knowledge/power configurations. Further still, the enactment of environmental modes of governing opens spaces of environmental governance and management across networks of actors and subjects. These include not only government and healthcare actors operating within state institutions but also extend to health and environmental activists and researchers, health industry representatives and individuals who seek to make informed healthcare choices within larger contexts and discourses of environmental regulation and sustainability.

While environmentality is a useful concept for focusing in on the knowledge/power nexus in the health sector’s environmental management, we do note that Foucault’s work on governmentality has long been criticised for its Eurocentricity (Legg, 2016), and a similar criticism can be applied here, including the fact that our own interpretations and reflections are informed by our work in the UK-based health sector. As such, we do not argue that this approach provides a comprehensive account of how the health sector is responding to environmental concerns. Nevertheless, drawing on the theoretical framework of environmentality allows us to bring attention to the nexus of power/knowledge that currently exists: by viewing the environment as socially constructed and by exploring how power/knowledge operates in this social construction, we can see how this nexus limits our thinking about how best to address environmental challenges. It limits our thinking by placing constraints on which knowledge can be produced to govern, how actors view this knowledge and through which lens, and actors’ ability to ask specific questions beyond these limitations. In the example of the green inhaler, we saw how focusing on carbon-centric approaches produced meanings of the environment that hid other environmental harms that then made them a site of *neglected* knowledge/power attention. Such neglected sites of knowledge/power hide present-day environmental harms and can also become problematic over time. Staying with the green inhaler example, Parker (forthcoming) describes how, at the time of the Montreal Protocol, hydro-fluoro-alkalines (HFAs) ozone depletion, not climate change, was seen as the more pressing issue; for this reason, HFAs were identified as a suitable alternative to the chloro-fluoro-carbon-containing metered-dose inhalers - the contribution of HFAs to global warming was something to be dealt with in the future.

Other neglected sites of knowledge/power exist. Revenge effects are a well-described example. As mentioned in the introduction, Tenner (1997) coined this term to name the unintended consequences that arise when a solution to solving a problem ends up making it worse. Increased efficiency measures to reduce carbon emissions are a good example of this: while increased efficiencies are viewed as an appropriate solution to addressing environmental harms, efficiency gains often rebound, leading to lower-than-predicted energy savings, and can even backfire, leading to consumption increases (Hilty et al. 2006, Widdicks et al. 2023). These effects rarely fit into the well-manicured emission spreadsheets that have clearly defined physical and virtual borders around which carbon-related data are deemed relevant for in/exclusion, and which emissions data are perceived as needing to be accounted for to show a reduction in consumption. In fact, such effects are likely purposely excluded in some instances, because they support more powerful knowledge/production sites associated with governance goals, such as an economic growth agenda (Widdicks et al. 2023).

Other sites of neglected knowledge/power include more diffuse and harder-to-capture understandings of the environment. Specifically, the entanglement of the health (and other) sectors’ environmental governance power/knowledge nexus, through spreadsheets and accounting practices, hides constructions of the environment that cannot be reconciled through such calculations, including those meanings of the environment not externalised from humans. Such meanings draw on deep ecology, indigenous, decolonial and other similar relational, planetary/one health and feminist perspectives to understand how humans, animals and the environment are interconnected (de La Bellacasa, 2017, The Lancet Public Health, 2022, Lehuedé, 2024).^20^ In these meanings, the environment is more than ‘out there’, and more than the air we breathe, the food we eat and beverages we drink; it is more than that which can be calculated. Rather, it is where ‘we live, work, and play’, the individuals with whom we interact, the buildings we live and work in, and even our own bodies (Pellow, 2017, Novotny, 1995). To understand these environments, understandings of embodiment and lived experience are needed, with attention to togetherness and intersectionality. In fact, understanding how individuals and communities manage their environments (their bodies, their spaces, their lives) can play a crucial role in contributing to environmentality in the health sector, helping to shift the knowledge/power nexus away from purely statistical knowledge/power generation. Returning to the green inhaler again, we might alternately study how individuals with respiratory illnesses manage their lived environments to reduce the likelihood of respiratory attacks, how broader society manages these environments, and the relationship between these forms of environmentality and those related to the health sector’s promotion of low-emission inhalers (Samuel et al. 2024). These aspects of environmentality are found in recent calls for respiratory illnesses to be managed through whole-systems approaches that improve local environments where people suffer from respiratory illnesses, moving away from self-governance to more top-down approaches (European Respiratory Society, 2021, Samuel et al. 2024).

Finally, meanings of the environment based on spreadsheet analyses can inadvertently lead to the de-prioritising of geopolitical social justice issues, for example, when accountancy (and accountability) remain associated with that which can be (or is chosen to be) calculated at a national level. Such calculative practices can hide environmental harms occurring outside of national jurisdictions, for example, when benchmarks and metrics in Europe, which are focused on emissions production within European boundaries, obscure emissions production (and broader social justice issues associated with this production) overseas which are moved outside of the purview of the Europe-based spreadsheet (Samuel et al. 2022).

## Conclusion

As our analysis has demonstrated, applying the concept of environmentality to the health sector has sensitised us to how new modes of governing through carbon-emission accounting, and other data practices and discourses, have been constructed by the health sector and internalised by researchers, patients and other actors. To illustrate what the lens of environmentality helps us see in the health context, we first discussed carbon accounting, describing how specific types of knowledge about carbon emissions are produced through various spreadsheets, conversion tables, data modelling, visualisation and accounting processes. Second, using the example of low-emission inhalers, we showed how this statistical framing of environmental knowledge has led to a power exercised through assigning patients the decision whether to switch to inhalers that are more ‘environmentally friendly’. We highlighted, as well, that these meanings and framings of the environment hide other environmental harms associated with the inhalers. In the final example, we examined how environmentality in health research operates beyond top-down modes of governance, in negotiations between health researchers and those who enact government. Reflecting on these examples, we conclude that environmentality can be conceptualised as a form of government, which has as its central objective the management and regulation of spaces and imaginaries that, held together, constitute ‘the environment’. Strategies and interventions to govern and regulate these spaces are guided and informed by configurations of knowledge/power, and are brought into being by the quantification of specific sources of data and the normalisation discourses that promote self-regulation and environmental responsibility.

Environmentality, as a concept, is thus an effective and productive vehicle in which to theorise, analyse and critique the changing trends of environmental governance in healthcare; to draw attention to regimes of knowledge, techniques and technologies that come into play at the interface of environmental management of the health sector; and to unearth and illuminate *neglected* sites of knowledge/power. As we have acknowledged in our analysis, healthcare infrastructures and ecosystems are vast, intricate and complex. As calls continue to be made globally regarding the environmental impact of healthcare systems and activities, further research utilising environmentality frameworks will be vital, not only for better understanding the interplay of knowledge/power across these sites but also for drawing attention and working to mitigate the unforeseen impacts of techniques of governance and management in the healthcare sector.

## Data Availability

No datasets were generated or analysed during the current study.

## Footnotes

1 https://www.legislation.gov.uk/ukpga/2008/27/contents

2 https://www.legislation.gov.uk/ukpga/2022/31/contents

3 Fletcher (2017: 312) also writes, ‘In addition to these, Foucault proposes, but does not further develop, the prospect of yet another “strictly, intrinsically, and autonomously socialist governmentality,” which, he claims, “is not hidden within socialism and its texts. It cannot be deduced from them. It must be invented” (2008: 94). These various governmentalities, Foucault proposes, now “overlap, lean on each other, challenge each other, and struggle with each other” (2008: 313)—an ongoing contest that, he suggests, is in fact what we commonly call “politics”’ (also see Foucault 2008).

4 There are many other ways that measure the environment, for example, the amount of plastic and other waste, the amount of resources used, etc, but carbon accounting is the predominant form of measurement.

5 https://www.ipcc-nggip.iges.or.jp/EFDB/main.php

6 An easy way to understand this is by considering the example of gender: if we categorise humans as male or female, we miss categories of non-binary genders, problematising the type of knowledge produced through this categorisation.

7 https://calculator.green-algorithms.org/

8 https://methodologyhubs.mrc.ac.uk/about/working-groups/trial-conductwg/tcwg-subgroup-greener-trials/

9 https://practicegreenhealth.org/tools-and-resources/health-care-emissions-impact-calculator#:~:text=The%20Health%20Care%20Emissions%20Impact,%2C%202%2C%20and%203%20emissions.

10 https://shcpathways.org/

11 https://greenhospitals.org/checkup

12 https://practicegreenhealth.org/tools-and-resources/health-care-emissions-impact-calculator

13 See, for example, https://igpp.org.uk/event/National-Sustainable-Healthcare-Conference-2024/; https://www.bsms.ac.uk/about/events/2024/06-25-share-conference.aspx; research for greener surgery conferences, and many more.

14 https://www.england.nhs.uk/nhs-commercial/sustainability/

15 https://www.legislation.gov.uk/ukpga/2022/31/contents; https://www.legislation.gov.uk/ukpga/2008/27/contents

16 https://www.greenerpractice.co.uk/information-and-resources/information-for-patients/inhalers/

17 https://www.nihr.ac.uk/documents/nihr-carbon-reduction-guidelines/21685#:~:text=The%20NIHR%20Carbon%20Reduction%20Guidelines%20are%20aimed%20at%20researchers%20conducting,on%20the%20associated%20carbon%20footprint.

18 The authors are aware of a number of surveys currently underway.

19 See previous note.

20 See https://www.who.int/health-topics/one-health#tab=tab1 and https://unfccc.int/climate-action/un-global-climate-action-awards/planetary-health
